# Digital Decision Support for Perioperative Care of Patients With Type 2 Diabetes: A Call to Action

**DOI:** 10.2196/70475

**Published:** 2025-04-08

**Authors:** Jianwen Cai, Peiyi Li, Weimin Li, Xuechao Hao, Sheyu Li, Tao Zhu

**Affiliations:** 1 Department of Anesthesiology, West China Hospital of Sichuan University, No. 17 Section 3 Renmin South Road, Chengdu, 610000, China, 86 18681357952; 2 Laboratory of Anesthesia and Critical Care Medicine, West China Hospital of Sichuan University, Chengdu, China; 3 The Research Units of West China (2018RU012)-Chinese Academy of Medical Sciences, West China Hospital of Sichuan University, Chengdu, China; 4 Department of Respiratory and Critical Care Medicine, West China Hospital of Sichuan University, Chengdu, China; 5 Institute of Respiratory Health, Frontiers Science Center for Disease-related Molecular Network, West China Hospital of Sichuan University, Chengdu, China; 6 State Key Laboratory of Respiratory Health and Multimorbidity, West China Hospital of Sichuan University, Chengdu, China; 7 Department of Endocrinology and Metabolism and Department of Guideline and Rapid Recommendation, Cochrane China Center, MAGIC China Center, Chinese Evidence-Based Medicine Center, West China Hospital of Sichuan University, Chengdu, China

**Keywords:** perioperative diabetes, artificial intelligence, clinical decision support systems

## Abstract

Type 2 diabetes mellitus affects over 500 million people globally, with 10%‐20% requiring surgery. Patients with diabetes are at increased risk for perioperative complications, including prolonged hospital stays and higher mortality, primarily due to perioperative hyperglycemia. Managing blood glucose during the perioperative period is challenging, and conventional monitoring is often inadequate to detect rapid fluctuations. Clinical decision support systems (CDSS) are emerging tools to improve perioperative diabetes management by providing real-time glucose data and medication recommendations. This viewpoint examines the role of CDSS in perioperative diabetes care, highlighting their benefits and limitations. CDSS can help manage blood glucose more effectively, preventing both hyperglycemia and hypoglycemia. However, technical and integration challenges, along with clinician acceptance, remain significant barriers.

## Impact of Type 2 Diabetes Mellitus in the Perioperative Period

Type 2 diabetes mellitus affects over 500 million individuals globally with 10%‐20% of these patients requiring surgery during hospitalization [1,2]. Throughout the whole perioperative period, patients with diabetes need more stringent blood glucose management, thorough complication evaluation, and multidisciplinary collaboration to mitigate mortality risk and enhance recovery, because diabetes is associated with an increased frequency of surgical interventions and prolonged hospital stays, with perioperative death rates 50% greater than those in the population without diabetes [3]. The contributing factors for these negative outcomes are multiple, but the main reason is perioperative hyperglycemia [4]. It can result in severe metabolic and organ dysfunction, exacerbate organ damage, trigger various disorders, increase infection risk, and even lead to postoperative death [5]. Although optimal glycemic control significantly improves postoperative outcomes in patients with diabetes, particularly in mitigating the risk of infection [6], there have long been obstacles regarding achieving the ideal method for managing blood glucose levels.

## Limitations of Current Perioperative Blood Glucose Management

Currently, perioperative blood glucose management is primarily categorized into three phases: preoperative assessment, intraoperative care and monitoring, and postoperative medication and diet [7]. Regular blood glucose monitoring during surgery is essential for effective perioperative control, often necessitating checks every 2 hours [8]. Nonetheless, stress responses, medication interventions, and several other circumstances can cause significant short-term elevations and rapid fluctuations in blood glucose levels [9]. Conventional measurement intervals are inadequate for detecting fast fluctuations in blood glucose levels and the cumulative impact of risk variables, thereby overlooking critical intervention chances. The American Diabetes Association’s Standard states that perioperative patients require more frequent blood glucose monitoring, particularly when insulin therapy is administered [10]. A 2-hour measurement interval may be insufficient for real-time control; thus, more frequent or continuous monitoring during surgery is recommended. In addition, blood glucose variability exposes patients to dual risks of hyperglycemia and hypoglycemia. Throughout this period, the Centre for Perioperative Care recommendations advise maintaining blood glucose levels between 6 and 12 mmol/L [1], contingent upon the administration of insulin and glucose, while either stringent or lenient blood glucose management may easily disrupt this “equilibrium.” Consequently, tools are required for real-time glucose data monitoring and individualized medication distribution [11].

## Potential of Clinical Decision Support Systems in Perioperative Blood Glucose Management

### Clinical Decision Support Systems

**Figure 1.**
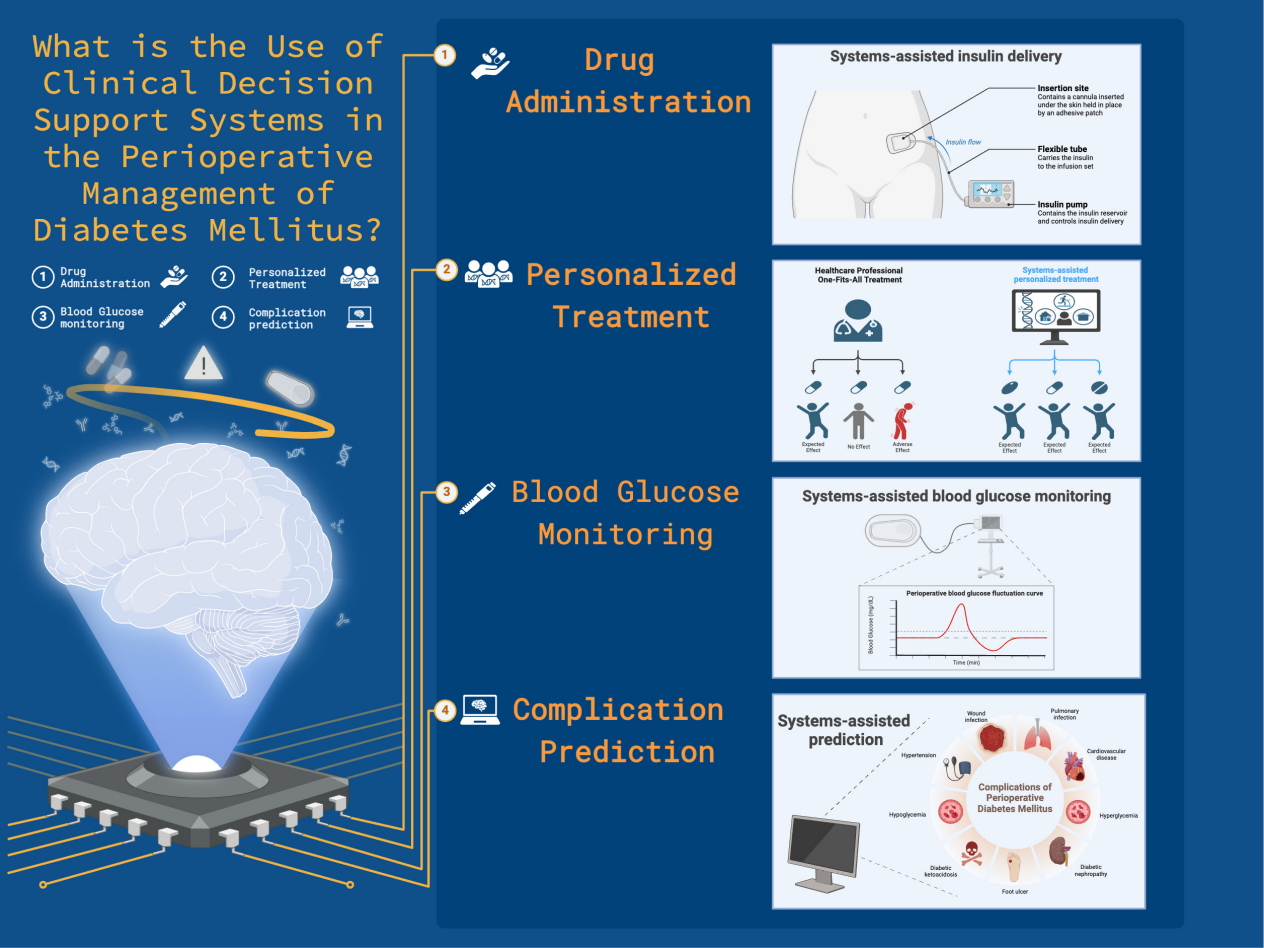
Application of clinical decision support systems in the perioperative care and management of diabetes mellitus.

Clinical decision support systems (CDSS) have gained significant traction due to the widespread adoption of electronic medical records and electronic health records in the past decades. These computerized systems may provide clinicians with a wide range of support, from basic pop-up warnings for medication errors to sophisticated tools that offer evidence-based recommendations for certain clinical situations (Figure 1) [12-14]. During the perioperative period, surgeons and anesthesiologists must consider multifaceted care, including blood glucose management, which requires experience and integrity in practice. The complexity of these tasks can be challenging for junior physicians and may create a gap between real-world clinical effectiveness and the efficacy observed in clinical trials. However, the advent of CDSS has introduced novel technical advancements to conventional perioperative management techniques.

A systematic review by Cai and colleagues summarizes trials and observational studies about the effectiveness of the CDSS in real-world settings [15]. As the American Association of Clinical Endocrinology stated in their 2023 Type 2 Diabetes Management Algorithm [16], personalized care is emphasized through evidence-based tools such as continuous glucose monitoring and automated insulin dosing systems. These technologies facilitated the ongoing surveillance of glucose levels and the secure delivery of insulin during surgical procedures. Furthermore, they have the potential to mitigate the likelihood of perioperative complications by assuring adherence to optimal glucose management guidelines [17]. Considering the aforementioned qualities, in contrast to conventional perioperative blood glucose control techniques, CDSS can address the challenge of detecting fast changes inherent in typical 2-hour monitoring intervals and provide real-time data to seize the critical intervention opportunities. Simultaneously, CDSS may precisely modify insulin dose according to real-time blood glucose data and specific patient situations, mitigating the twin hazards of hyperglycemia and hypoglycemia, thereby facilitating more effective and safer blood glucose regulation.

### Personalized Drug Delivery

Glucommander (Glytec) has set an example as an electronic glycemic management system since 1984 using a computer-based algorithm to guide the administration of intravenous insulin [18]. Glucommander (Glytec) formulates insulin dosage recommendations by analyzing patient-specific blood glucose patterns after health care professionals choose either a personalized dosage or a weight-based multiplier as the first dosing approach for the first 24 hours [19]. Until now, after undergoing integration and evaluation at multiple medical centers, Glucommander (Glytec) has demonstrated its effectiveness in enhancing outcomes for surgical patients with both type 1 and type 2 diabetes. Specifically, it has reduced the occurrence of hyperglycemia and hypoglycemia, treated diabetes ketoacidosis, and increased adherence to the guidelines for achieving individualized treatment [20-22].

### Intraoperative Blood Glucose Level Monitoring

Similarly, there are CDSS that continuously monitor changes in perioperative blood glucose levels by providing in-room pop-up prompts. In a systematic review [15], one of the included studies [23] designed and evaluated a new CDSS using Epic’s best practice advisory (BPA) framework. This tool is designed to remind anesthesia providers to measure blood glucose levels at specified intervals for patients at risk of abnormal perioperative blood glucose levels. The research results found that the implementation of the BPA CDSS significantly improved intraoperative blood glucose monitoring and management in the postanesthesia care unit (PACU). The PACU hyperglycemia rate decreased from no CDSS to the BPA CDSS (10.4% to 7.2%, *P*=.031).

## Dispersion and Untimely Integration of Data Affects the Functionality of CDSS

### Barriers Hindering the Effectiveness of CDSS

Notably, several barriers currently hinder the effectiveness of CDSS, highlighting the call for action. For patients with type 2 diabetes during the perioperative period, multimodal data are necessary for the development and maintenance of an effective CDSS, such as blood glucose monitoring data, drug information, surgical types, comorbidity, and more. However, such data are often scattered across various systems and require manual input. The integration of a substantial volume of data from many systems and the necessity for real-time updates in CDSS impose significant requirements on technical and system compatibility attributes. Moreover, blood glucose levels change continuously throughout the whole perioperative period caused by surgery, stress, or fasting. Without timely integration, decisions may be delayed or based on outdated information, thereby adversely impacting patient outcomes. Insufficient integration between electronic health records and CDSS might compromise the real-time prediction and accuracy of critical data [24].

### Alarm Fatigue and Clinician Skepticism

CDSS-based decisions combine data, algorithms, clinicians’ expertise, and clinical judgment. An estimated 95% of CDSS alerts were declined by clinicians [25]. The sheer volume of redundant messages exacerbates the burden in the practice. Some clinicians may develop “alarm fatigue” and become desensitized to all warnings, including those that are clinically valuable. Nonetheless, placing excessive reliance on CDSS recommendations is not always appropriate. Despite the exceptional accuracy of the generated data, CDSS are fundamentally an opaque system with an internal operational mechanism that is hard to interpret [26]. If professionals simply press the button of CDSS without comprehending the underlying principles, such decisions will be very dangerous. Assuming that patients with diabetes mellitus experience hypoglycemia and hyperglycemia crises, professionals should not only be proficient in how to obtain emergency care advice through CDSS, but also implement appropriate care measures based on their own experience and understanding of patient data. Therefore, what stands out the most is striking a balance between CDSS and clinicians, or in other words, algorithms and clinical experience of diabetes management.

### Balancing Cost-Effectiveness

The development and maintenance of CDSS require acknowledging the need for robust data sources, advanced informatics systems, technical support, and personnel training. Building CDSS from the ground up to meet criteria can incur substantial costs, ranging from hundreds of thousands to millions of dollars. Custom-developed systems also require ongoing maintenance and upgrades. Maintenance expense usually varies from 10% to 20% of the original development expenditure. The annual cost of maintaining CDSS for diabetes management is approximately US $9500 for one small-sized institute, US $20,600 for medium-sized, and US $76,000 for large-sized ones [27]. For medical institutions with limited resources, managers need to weigh whether the potential improvements in patient outcomes or compliance with perioperative medical personnel guidelines are worth the high cost [28]. After rigorous evaluation, the effectiveness of some CDSS has been found to be disappointing. Jeffery and colleagues [29] systematically reviewed 15 randomized trials that assessed the effectiveness of CDSS in diabetes mellitus management compared with a non-CDSS control group (usual care, seminars, educational material, and glucose monitoring systems), but found no significant outcome that CDSS could reduce hospitalizations and improve quality of life. Meanwhile, the study found that in the third month, the pooled estimate of the change in glycated hemoglobin (HbA_1c_) was 5 mmol/mol (95% CI –9 to 1; ie, –0.5%, 95% CI –1.0 to 0.1), but it is only a clinically significant threshold and this result is not significant. Blindly using CDSS may result in getting half the results with double the effort.

### Future Direction

The number of CDSS specifically designed for diabetes management remains limited. The majority of CDSS development is directed towards traditional perioperative patients, emphasizing factors such as surgical type, patient age, and vital signs; however, limited attention is given to comorbidities, such as diabetes, in patients undergoing surgery. A prospective study in 29 countries across Europe identified diabetes mellitus as the fourth most common long-term condition (15.4%). Meanwhile, diabetes mellitus also accounts for a large share of patients with multimorbidity, with 19.4% of patients having two long-term health conditions and 43.8% having more than three long-term health conditions [30]. The coexistence of multiple diseases substantially elevates the mortality rate of patients undergoing surgery, sometimes doubling it. Poorly controlled chronic diseases, such as those with high American Society of Anesthesiologists scores, and compromised functional status (eg, frailty) further heighten these risks. If the database used for developing CDSS does not cover specific patients (such as those with complex comorbidities), this deficiency may lead to the system ignoring the risk factors of specific patients and providing treatment recommendations with biased risks.

The disparity in diabetes care throughout the world is becoming worse. The treatment rate of diabetes has remained low and relatively unchanged for many low-income and middle-income nations during the last several decades. More than 90% of people with diabetes in some nations did not get treatment between 1990 and 2022 [31]. Limited by the ratio of doctors-to-patients and infrastructure, diabetes may impose a heavier burden on these low-resource clinical environments, which may require the introduction of CDSS. However, its effectiveness in the low-resource environment remains to be explored [32]. In the presently advanced CDSS applications, the initial datasets used for development mostly originate from populations in developed countries, and their efficacy is often poor when applied to other locations or populations. A skin cancer diagnostic algorithm developed using data from White patients may have reduced efficacy for those with darker skin tones [33]. Prior to implementing these systems, it is essential to analyze data bias to avert unjust decision-making and mitigate health disparities among ethnic minorities or resource-limited regions. Furthermore, the deployment of CDSS necessitates the integration of information systems and financial investment, taking into account the restricted accessibility and technical capacities in resource-constrained regions. This has prompted demands for international collaboration, including the implementation of remote medical platforms or the direct supply of digital medical assistance [34].

In light of the issues faced by present CDSS implementations, the following recommendations are anticipated to be implemented (Table 1). Initially, at the source, deep learning techniques may be used to extract unstructured data from multimodal text and combine it into a unified system for analysis after standardization [35]. This metric enhances both the frequency of CDSS use and its real-time performance [36]. A potential innovation is the digital twin, a mathematical model of a system created from all accessible data. This technique may generate a virtual personal twin of a perioperative patient, capture the patient’s perioperative trajectory without affecting their physiological condition, and be used for complication prevention and rehabilitation treatment [37]. Secondly, given that clinical physicians’ adoption of CDSS and alarm fatigue may arise from their skepticism towards the system and their proficiency in computer abilities, it is essential to investigate their requirements and formulate targeted training programs during the design phase of CDSS [36]. The alarm system may be enhanced by applying human factors engineering principles, hence minimizing false alarms and overlooked alerts [38]. Lastly, a systematic, step-by-step strategy is essential. It is advisable to do feasibility studies and pilot studies prior to real-world implementation, not only to identify software and hardware difficulties during the deployment phase but also to assess the long-term cost-effectiveness across various health care settings [39].

**Table 1.** Functions of clinical decision support systems (CDSS), limitations, and evidence-based solutions.

Functions of CDSS	Limitation of CDSS	Solutions to break limitations	Explanation of solutions
**Personalized Drug Delivery** Based on real-time blood glucose data and the patient’s specific condition to calculate and recommend the appropriate insulin dosage.	**Data Integration Defects** The data required for CDSS are usually scattered across various systems and require manual input, which affect the real-time performance of analysis.	Integrate the required data for analysis into the same system adopting new technologies.	Deep learning techniques can extract and analyze relevant unstructured information from clinical records, including single concept extraction, temporal event extraction，relation extraction，and abbreviation expansion [35].
**Blood Glucose Monitoring** Real-time monitor changes in perioperative blood glucose levels by providing in-room pop-up prompts.	**Alarm Fatigue** Too many unnecessary alerts or suggestions lead to providers losing trust or being insensitive to CDSS.	Applying human factors engineering principles to design the alarm systems.	A system designed based on human factors principles may alleviate alarm fatigue, with specific strategies including reducing errors related to availability, delivering clinical data nearer to the decision point, and presenting alert text in a tabular style [38].
**Blood Glucose Management** Adherence to clinical guidelines to perform clinical procedures.	**Clinician Skepticism** Clinicians have a resistant or opposing attitude towards the opinions or suggestions given by CDSS.	Consider the needs of clinicians and develop specific training plans.	Clinicians should be involved in the design and development of CDSS in the early stages, and receive hands-on training and education before implementation. Clinicians’ negative attitudes and resistance towards CDSS can be alleviated during this process [36].
**Complication Prediction** Utilizing patient perioperative information to predict the incidence of adverse events (such as hyperglycemia, hypoglycemia, etc)	**Cost Challenges** The development and maintenance of CDSS may consume capital or human resources and cannot guarantee long-term cost-effectiveness.	Do feasibility studies and pilot studies prior to real-world implementation. Long term follow-up to collect cost-effectiveness data.	Feasibility studies and pilot studies can help determine whether CDSS can transfer its good performance from the development phase to real-world settings, ensuring its correct and safe use in health care practice [39]. In addition to collecting cost data, long-term indicators such as patient prognosis or quality-adjusted life years should also be collected to determine whether the implementation of CDSS is a good return on investment for both hospitals and patients [28].

## Conclusion

In summary, CDSS are revolutionizing the paradigm of perioperative diabetes mellitus care and management in the real world, shifting from conventional strategies to data-driven real-time monitoring and individualized treatment. Given the high volume of surgeries for patients with diabetes and the elevated incidence of postoperative complications, these systems are promising in many ways: integrating patients’ blood glucose monitoring data and providing real-time blood glucose fluctuation warnings, offering personalized medication recommendations to prevent drug interactions or improper dosage adjustments, and assisting health care providers in predicting the surgical risk based on the patient’s historical data (HbA_1c_, preoperative blood glucose control, complications, and other factors). However, several barriers currently hinder the effectiveness of CDSS, though the original intention of these intelligent health intervention measures is to address the existing difficulties in the management and care of perioperative patients with diabetes mellitus. Thus, future research on CDSS must prioritize model optimization, particularly enhancing performance for patients with intricate comorbidities, especially diabetes, and develop techniques to bolster physicians’ confidence and acceptance. Several randomized controlled trials and cost-benefit analyses with extended follow-up durations across various countries to validate the system’s efficacy, universality, and practicality, and a pilot study are recommended before implementation. This will ultimately ensure that the system can cover high-risk factors and provide evidence-based treatment recommendations, reducing the worldwide diabetes care disparity and advancing health equality.

## Abbreviations

BPA: best practice advisory
CDSS: clinical decision support systems
HbA_1c_: glycated hemoglobin
PACU: postanesthesia care unit
